# Obstetrics

**Published:** 1876-01

**Authors:** 


### Summary of Progress in the Medical Sciences.

### I. Obstetrics.

1. *On the Management of the Lying-in-Woman.* Hime. (*Obstetric. Jour. of Great Britain and Ireland*, Oct.)

The old theory, which represents the lying-in-woman as being in a state, similar to that of a person after an amputation, the uterus being compared to the part operated on, is unscientific and untenable. Parturition is a physiological process—the fulfillment of a natural function, and has no analogy with an operation which is an interference with function. Amputation, whether the result of disease or accident, involves consequences which have no analogue in the process of parturition. The uterus after labor is no more comparable to a stump after amputation, than the uterus after or during menstruation. After natural labor there is nothing comparable to the collapse succeeding a major amputation ; there is no fever, no suppression of secretions, no suppuration, or, if pus be present, it is not derived from the uterus at all, but from the vagina or external genitals, in the great majority of cases. The insignificant rise in temperature from 0.5° C. to 0.8° C. (the former in multiparæ, the latter in primiparæ) is due to normal physiological and not to morbid action, being the effect of muscular exertion, increased activity of the lungs, liver and other organs, when relieved from the pressure of the gravid uterus; and is only fleeting. Milk fever is far more talked of and written about than seen, and is of rare occurrence. The rise of temperature which accompanies the commencement of mammary activity, is slight, temporary, and unaccompanied by mental depression or constitutional disturbance of any kind. Operations performed immediately after labor will yield kindly.

A decided alteration, then, is needful in the common mode of treating lying-in-women as patients—confining them to bed for ten or twelve days on a low diet—the ordinary puerperal dietary being such as would certainly not be given to any patient after amputation. Water gruel, barley water, tea and dry toast should be abandoned for milk, eggs, good soup, chickens and other digestible meats, to be given from the first, and, of course, in quantities suitable to the conditions of individuality, want of exercise, etc. Stimulants are decidedly injurious, except in special cases. It is often urged, that, as a large amount of waste uterine tissue has to be got rid of, low diet should be adhered to ; but milk has also to be secreted, and, anyhow, health and vigor will promote excretion and the performance of all vital functions better than a state of debility. Opiates, ergot and other drugs should only be given under necessity. The child should be applied as soon as the mother's state permits ; if there be no milk at first, only for a moment or so, to encourage its secretion and the involution of the uterus. The binder is more of an *euthanasia* than a benefit after the first twelve hours, but not so the early removal into a fresh bed and room, if possible, and this may be done within forty-eight hours. The woman may sit up in bed for a short time from the first, a continual maintenance of the recumbent posture for ten or twelve days being as injurious as it is unnecessary; and most patients may be on the sofa on the fourth or fifth day. Above all things, the medical attendant should see that his directions are carried out, and not trust they will be so, especially as to the removal of soiled linen, etc.; not that its presence, any more than the neighborhood of privies, want of ventilation, etc., will, *per se*, develop metria any more than typhoid fever: otherwise eight or nine-tenths of lying-in-women must inevitably suffer from it, a result equally certain if medical men could convey the germs of disease with them as readily as is assumed. Cleanliness and ventilation always tend to preserve health and check disease, but they are no more needful for the lying-in-woman than nourishing food. After natural labor a woman is not in a diseased state, and the maintenance of health and vigor will be the most successful means of averting all risks.

2. *Management of Third Stage of Labor.* WALLACE. (*Obs. Jour. of Great Britain and Ireland*, Oct. 15.)

The position of the placenta is most frequently on the middle zone of the uterus, next on the fundal zone. According to its position is the mechanism of expulsion. Smellie and others knew the true mechanism of the expulsion and delivery of the placenta, which has recently been brought to the notice of the profession by Matthews Duncan, Lemser, Caseaux, Leishman, and others, in contradistinction to the descriptions given by Baudelocque, Schultze, etc. The routine practice of the binder, ergot, opium and the cordials after delivery, was condemned. It was especially shown that the binder, as applied with pads, converted an abdominal organ, as is the uterus at that stage, into a pelvic one, and hence caused, instead of prevented, bleeding; and was one of the main factors in producing subsequent uterine trouble, in the way of flexions and displacements.

3. *Medico-Legal Aspect of Abortion.* LEBLONDE. (*Annales de Gynécologie*, Aug., 1875.)

From eleven cases the author deduces the medico-legal value of the integrity of membranes in abortion occurring during the early months of pregnancy.

(*a.*) When abortion occurs "en bloc" (embryo within sound, unbroken membranes), it is probably spontaneous; or, at least, not produced by agents which determine the expulsion of the ovum without implicating the membranes.

(*b.*) When the membranes are ruptured, but healthy, in all probability abortion has been provoked.

(*c.*) When the membranes are pathologically altered, no conclusion can be derived from the expelled product, though spontaneous abortion has probably resulted from disease of the ovum.

